# The prevalence of nomophobia in Cyprus and its relationship with coping styles

**DOI:** 10.3389/fpsyg.2025.1538155

**Published:** 2025-03-05

**Authors:** Marilena Mousoulidou, Erietta Constantinidou, Andri Christodoulou, Michailina Siakalli

**Affiliations:** Department of Psychology, Neapolis University Pafos, Paphos, Cyprus

**Keywords:** nomophobia, Cyprus, coping styles, smartphones, avoidance, social media

## Abstract

**Introduction:**

The rise in smartphone use and the resulting dependence has led to the emergence of nomophobia, a term describing the anxiety or discomfort experienced when individuals are without their mobile phones. This study aimed to examine the prevalence of nomophobia among adults in Cyprus and explore its relationship with demographic characteristics, reasons for smartphone use, and coping styles.

**Methods:**

In line with this aim, participants were 300 adults from Cyprus recruited by convenience and snowball sampling methods. The data were collected via an internet-based questionnaire that examined participants' level of nomophobia, reasons for phone use, time spent on their phones, and coping styles. The study utilized a Personal Information Form, the Nomophobia Questionnaire (NMP-Q), and the Brief Coping to Problems Experienced Inventory (Brief COPE) to gather data.

**Results:**

The results suggest that (a) nearly all participants (99.3%) exhibited some level of nomophobia, with more than half of our sample (51.3%) experiencing moderate levels, (b) younger adults, women, and individuals with lower education levels were more prone to nomophobia, (c) communication and social media were positively related to nomophobia, and (d) maladaptive and avoidant coping strategies exacerbated the severity of nomophobia.

**Discussion:**

The findings highlight the growing concern of nomophobia and stress the need for educational programs promoting healthier smartphone habits.

## 1 Introduction

The invention of mobile phones is one of the most significant technological breakthroughs in human history (Harris and Cooper, 2019). Nowadays, the use of mobile phones has risen considerably, leading to a dramatic increase in the usage of well-known smartphones, which have become an essential part of modern life (Panova and Carbonell, 2018). The number of mobile users worldwide has increased from 7.1 billion in 2021 to 7.26 billion in 2022, and it is expected to reach 7.41 billion and 7.49 billion in 2024 and 2025, respectively (Statista, 2023). Smartphones provide functions such as information search, study, social contacts, games, and entertainment (Jeong et al., 2016), thus becoming more essential and useful to their users and an inseparable element of daily life (Gnardellis et al., 2023b). Undoubtedly, using social media and social network sites, along with the ease and speed with which information and other online services are accessed, has several positive effects. Adding to this is medical aid through smartphone-based applications, which enable people to perform some aspects of their jobs faster and more efficiently (Bragazzi and Del Puente, 2014). Nowadays, both communication and information are just a “click” away, making life much more accessible for people (Gonçalves et al., 2020). This range of capabilities resulted in the widespread acceptance of smartphones (Gnardellis et al., 2023b), becoming a necessary component of modern life (Bragazzi and Del Puente, 2014).

Clearly, the introduction of smartphones has caused a considerable change in people's daily lives. Beyond the positive aspects, however, there are several adverse physical effects, such as musculoskeletal issues and pain (Mohani et al., 2024), headache, neck pain, spine issues, and sleep problems (Daraj et al., 2023; Hamutoglu et al., 2018; Kurnia et al., 2021; Notara et al., 2023; Oraison and Wilson, 2024; Singh and Rathore, 2023). Psychological and behavioral changes have also been reported (Rodríguez-García et al., 2020). One of the primary adverse effects of smartphone use is technology addiction, with the most prevalent types being internet addiction, social media addiction, internet gaming disorder, and smartphone addiction (Singh and Rathore, 2023). Within this context, research into smartphone addiction led to the emergence of a relatively new term known as nomophobia.

Nomophobia refers to the discomfort or anxiety that someone feels when his/her mobile phone is not available, and it is defined as the fear that a person experiences, which is related to the lack of use or even access to their smartphone (Bulut and Sengul, 2024). Worth noting is that the wide acceptance and importance of this new term has led researchers to propose that nomophobia should be included in the Diagnostic and Statistical Manual of Mental Disorders (DSM; Bragazzi and Del Puente, 2014; Kaviani et al., 2020), in some cases under the name of “Smartphone Addiction Disorder” (Oraison and Wilson, 2024). According to Galhardo et al. (2020), nomophobia can be considered a situational modern-age phobia. It can be expressed by individuals with nomophobia through feelings of worry and anxiety when they have no access to their mobile phone for any reason, obsessive control of it even when they have it next to them, avoidance of places where mobile phones are not allowed, permanent carrier of a charger to ensure their phone will not run out of battery, and keeping their phones on and next to them during the night (Hamutoglu et al., 2018). It is suggested that the symptoms of nomophobia may create the need for the investigation of the presence of a possible pre-existing mental disorder, which may be diagnosed and treated (King et al., 2013).

The definition of nomophobia highlights psychological dependence and the modern fear and anxiety related to mobile phone use, specifically when being without a mobile phone, unable to use it or communicate, and losing access to information (Yildirim and Correia, 2015). While many definitions seem to associate the unavailability of mobile phones with feelings of anxiety, nomophobia is also considered a situational phobia, which is related to agoraphobia (King et al., 2010). Additionally, the fear of missing out (FoMO) has been identified as a major contributor to nomophobia (Ceobanu et al., 2023). It is also worth noting that attachment theory has been proposed as a theoretical framework for understanding nomophobia, suggesting that it shares characteristics with separation anxiety, as losing access to mobile phones may trigger anxiety responses similar to those observed in separation anxiety within close relationships (Han et al., 2017).

Several countries have examined the prevalence of nomophobia. For instance, in Italy, almost 50% of the participants exhibited at least moderate nomophobia (Bragazzi et al., 2019). Findings from Australia suggest that 99.2% of the participants showed some level of nomophobia, and of those, 48.7% and 13.2% were classified as having moderate and severe levels of nomophobia, respectively (Kaviani et al., 2020). In Croatia, moderately high levels of nomophobia have been observed (Santl et al., 2022), and significant levels of nomophobia have also been found in India, the Philippines, Tunisia, and Malaysia (Al-Mamun et al., 2023; Anjana et al., 2021; Ferchichi et al., 2023; Oraison and Wilson, 2024; Saleh et al., 2019). A meta-analysis of 2023 showed that the prevalence of moderate to severe nomophobia was high, reaching 70.76% (Al-Mamun et al., 2023). It is also worth noting that the prevalence of nomophobia has increased during the COVID-19 home confinement (Aydin and Kuş, 2023).

Existing literature that examined the relationship between nomophobia and several physical and mental problems has shown statistically significant relations (Rodríguez-García et al., 2020). In general, a positive relationship was observed between nomophobia and adverse health status (Anjana et al., 2021; Notara et al., 2023). Specifically, studies have shown a significant positive relation between social phobia (Tárrega-Piquer et al., 2023), separation anxiety, total anxiety, depression, and nomophobia (Kuscu et al., 2021).

Nomophobia was also closely related to low self-esteem (Vagka et al., 2023a; Valenti et al., 2022), smartphone addiction (Al-Mamun et al., 2023; Daraj et al., 2023; Oraison and Wilson, 2024), insomnia symptoms, and poor sleep quality (Daraj et al., 2023; Kurnia et al., 2021), depression, stress and anxiety (Çakmak Tolan and Karahan, 2022; Gnardellis et al., 2023b; Santl et al., 2022; Singh and Rathore, 2023), and the fear of missing out (FoMO; Hamutoglu et al., 2018; Wen et al., 2023). Emotional exhaustion at work (Wang and Suh, 2018) and poor academic performance (Gutiérrez-Puertas et al., 2019; Wen et al., 2023) have also been found by previous research to be positively related to nomophobia. Finally, research has shown that shyness, anxiety, and loneliness can positively predict nomophobia, explaining 36% of the variance of nomophobia scores (Dehghanian and Bordbar, 2023; Valenti et al., 2022; Zhang et al., 2023).

Looking at the relationship between age and nomophobia, studies suggest that smartphone addiction and nomophobia scores are significantly higher in younger ages than in older people. More specifically, age level is negatively related to nomophobia levels (Guimarães et al., 2022; Guzel and Alan, 2020; Kaviani et al., 2020; Vagka et al., 2023b). Nevertheless, results are inconclusive since some studies suggest no significant relation between nomophobia and age (Adawi et al., 2018; Galhardo et al., 2020; Saleh et al., 2019). An important finding in this line of research is that there appears to be a negative relationship between nomophobia level and the age at which a person uses a smartphone for the first time (Bulut and Sengul, 2024).

Regarding gender differences, a significant finding is that women not only exhibit higher levels of nomophobia than men, but they are also more likely to develop a severe level of nomophobia instead of mild or moderate levels (Galhardo et al., 2020; Kaviani et al., 2020; Moreno-Guerrero et al., 2020; Vagka et al., 2023b). Since nomophobia is under the umbrella of specific phobias, it is worth noting that studies that examined gender differences in specific phobias showed that specific phobia levels are higher in females than in males (Bourdon et al., 1988; Fredrikson et al., 1996; Settineri et al., 2019; Wardenaar et al., 2017), which supports this finding. Lastly, a systematic literature review showed that nomophobia was more prevalent among young adults and women rather than older adults and men (Notara et al., 2021). Note, however, that there are also studies in the literature that suggest that men exhibit higher levels of nomophobia or smartphone addiction (Guzel and Alan, 2020; Oraison and Wilson, 2024), whereas (Saleh et al., 2019), revealed no significant differences among the two genders. Therefore, future studies need to explore this finding further.

As far as mobile phone usage and nomophobia are concerned, social network services were found to be a stronger predictor of mobile phone addiction than game use (Jeong et al., 2016). Furthermore, social media habits have been found to significantly predict nomophobia (Okur et al., 2022). The finding of another study revealed that higher rates of smartphone use for social media were reported by participants with severe nomophobia (Vagka et al., 2024).

Coping styles can assist individuals in managing phobias. Coping styles or strategies refer to the specific behavioral, cognitive, or psychological adjustment mechanisms and skills people use to deal with, counteract, or overcome a stressful event (Bragazzi et al., 2019; Yusoff, 2010). Coping is considered to be of critical importance both to physical and psychological health for people experiencing stressful situations (Kapsou et al., 2010). Coping strategies were classified by previous research work in two different ways. Some research classified them as emotion-focused, problem-focused, and avoidance strategies, and others as adaptive and maladaptive coping strategies (Bai et al., 2020). The literature that examined the relationship between nomophobia and coping styles has demonstrated some interesting findings. Specifically, the study of Bragazzi et al. (2019), showed that people with higher nomophobia scores were more likely to adopt maladaptive coping strategies such as denial, self-distraction, self-blame, behavioral disengagement, and venting. A study carried out in India showed that most college students with mild and moderate nomophobia were adopting the problem-focused coping style. In contrast, students with severe nomophobia were mainly using the emotion-focused strategy (Gupta et al., 2024). Finally, a study that examined the relationship between mobile phone addiction and positive or negative coping styles showed that mobile phone addiction was positively associated with a negative coping style (Lu et al., 2021). This is an important finding considering that mobile phone addiction is closely related to nomophobia (Yildiz Durak, 2019). The lack of extensive research examining nomophobia through the lens of coping styles, together with the unclear and inconclusive results of the existing literature, are the reasons for including coping strategies as variables to be examined in the current study.

The current study aims to explore the prevalence of nomophobia among adults in Cyprus. Specifically, it examines nomophobia's relationship with demographic characteristics, such as age and gender, phone usage, and coping styles. To the best of our knowledge, nomophobia has not been previously studied in Cyprus. An exception to this is a recent study conducted in Greece with a Greek-speaking population, which showed that 99.9% of the participants exhibited some level of nomophobia, with moderate nomophobia at 57% and severe at 18.9% (Vagka et al., 2023b). Furthermore, current research in other countries shows that increased smartphone use and the consequent rise in nomophobia pose an emerging threat to mental health (Notara et al., 2021). Although no prior research specifically investigates nomophobia in Cyprus, smartphone statistics highlight a rising trend. In 2024, the revenue of the Cypriot smartphone reached US$95.3 million, with an annual projected growth rate of 2.22% from 2024 to 2029 (Statista, 2023, 2024). By comparison, China generated the highest revenue globally, with US$105.5 billion in 2024. Per capita, Cyprus generated US$76.95 in 2024, and the smartphone market volume is expected to reach 0.4 million units by 2029, with an estimated growth of 1.1% in 2025. In 2024, Cyprus's average smartphone ownership rate is projected at 0.3 units per person. This increasing demand for smartphones in Cyprus, which exceeds the EU average, suggests a tech-savvy population with rising smartphone adoption (CyprusMail, 2021). The high usage rates may also signal potential phone addiction, which is closely related to nomophobia. Consequently, high levels of nomophobia may also be revealed in Cyprus, underscoring the urgent need to investigate nomophobia in Cyprus. Based on the existing literature and the purpose of the study, the following hypotheses were formulated:

**Hypothesis 1 (H1)**. The prevalence of nomophobia in the current sample is expected to be high. Since no studies have been conducted in Cyprus, this hypothesis was exploratory. However, existing literature from other countries, particularly Greece where the population shares a common language with Cyprus, suggests that nomophobia levels are likely to be high.

**Hypothesis 2 (H2)**. There will be associations between demographics and nomophobia levels. Based on the existing literature, younger individuals are expected to display higher levels of nomophobia than older individuals. Regarding gender differences, the literature remains inconclusive on whether women exhibit higher levels of nomophobia than men; therefore, this study explores whether such differences exist in the current sample. Additionally, the current study examines whether education level might influence nomophobia levels, a factor that has not been examined in past research.

**Hypothesis 3 (H3)**. There will be associations between the reasons for phone usage and nomophobia. Previous literature has shown a relationship between social media usage and nomophobia. Since social media is the predominant digital activity globally, it is expected to correlate positively with nomophobia.

**Hypothesis 4 (H4)**. There will be associations between coping styles and nomophobia. Even though research in this area is limited, available studies suggest that a problem-focused coping style will likely be negatively related to nomophobia. In contrast, an avoidance coping style will be positively related. Additionally, considering the distinction between adaptive and maladaptive coping strategies, it is expected that maladaptive coping strategies will be positively associated with nomophobia.

## 2 Materials and methods

### 2.1 Procedure

Upon the necessary approval from the Cyprus National Bioethics Committee (EEBK EΠ 2024.01.94), the measures used in this study were compiled into a single questionnaire created via Google Forms. Data were collected via convenience and snowball sampling methods between the 10th and the 29th of April 2024 to recruit as many participants as possible. The authors distributed invitation calls to participate in the study via social media (e.g., Facebook) and social chatting apps (e.g., Viber, WhatsApp, Messenger) and emailed them to personal contacts. All participants were informed about the study's purpose and duration and assured of their anonymity when participating. Consent to participate was obtained before completing the questionnaire, which took approximately 10 min. The survey remained online for 3 weeks until the required sample of 300 participants was secured.

### 2.2 Sample

Eligibility for participation in this study was limited to residents of Cyprus aged 18 years and over. A review of the responses confirmed that no participants were under the age of 18. Additionally, all participants were native Greek speakers residing in mainland Cyprus. The current study's final sample consisted of 300 adult participants. Of these, 205 were female (68.4%), 94 were male (31.3%), and one identified as other (0.3%). Participants' ages ranged from 18 to 77, with a mean age of 34.9 years (SD = 13.8 years). One hundred and three of the participants (34.33%) were emerging adults (18–25 years old), 90 participants (30%) were early-aged adults (26–39 years old), and 107 participants (35.67%) were middle-and-late-aged adults (40–77 years old). Regarding education level, 80 participants (26.7%) completed secondary education, 117 participants (39%) finished their bachelor's degree, 95 participants (31.7%) completed their master's degree, and 8 participants (2.6%) finished their doctorate degrees.

### 2.3 Measures

The data were collected through (I) the Personal Information Form, (II) the Nomophobia Questionnaire (NMP-Q) developed by Yildirim and Correia (2015), and (III) the Brief Cope Questionnaire (Brief COPE) developed by Carver (1997).

#### 2.3.1 Personal information form

The form was developed by the researchers. It included questions to gather demographic characteristics on participants' age, gender, and educational level. In addition, two questions were developed and administered to examine the characteristics of our sample regarding mobile use. Question 1 asked participants to state the reasons for using their mobile phones. Participants were given six options and were able to select multiple answers. The options were: (a) communication, (b) social media (e.g., Facebook, Instagram, Twitter), (c) information (e.g., newspapers, scientific journals), (d) professional reasons (e.g., online meetings, emails), (e) games, and (f) other reasons. The second question asked participants to indicate how much time they spend on each of the above reasons (i.e., communication, social media, information, professional reasons, games, and other reasons). This question used Likert-type options, allowing participants to choose from the following time categories: (a) none, (b) 1 h, (c) 2 h, (d) 3 h, and (e) more than 3 h.

#### 2.3.2 Nomophobia questionnaire

To explore participants' nomophobia level, the 20-item Nomophobia Questionnaire (NMP-Q) originally developed by Yildirim and Correia (2015) and validated in the Greek language by Gnardellis et al. (2023a) was employed. The questionnaire includes 20 questions (e.g., If I did not have my smartphone with me, I would be worried because my family and/or friends could not reach me), which are rated on a Seven-point Likert-type scale, ranging from 1 (strongly disagree) to 7 (strongly agree). The sum score of the participants' responses ranges between 20 and 140, with higher scores indicating more severe levels of nomophobia. Specifically, the sum score divides participants into four levels of nomophobia: (a) absence of nomophobia (score between 0 and 20), (b) mild nomophobia (score between 21 and 59), (c) moderate nomophobia (score between 60 and 99), and severe nomophobia (score between 100 and 140). Additionally, the questionnaire not only measures the general level of nomophobia but also categorizes it into four factors, which provide a qualitative understanding of the condition. Factor 1, “not being able to communicate,” includes six items and corresponds to the anxiety individuals feel when they are unable to connect with others. Factor 2, “losing connectedness,” comprises five items and refers to individuals' fear of being disconnected from social networks and online communities. Factor 3, “not being able to access information,” includes four items and corresponds to the anxiety individuals feel about not having access to information. Factor 4, “giving up convenience,” comprises five items and responds to individuals' discomfort from losing the convenience of using apps (Galhardo et al., 2020). The items' internal reliability for the current sample was excellent, with a Cronbach's alpha value of 0.945. The reliability of each factor was also high; Factor 1: α = 0.951, Factor 2: α = 0.9, Factor 3: α = 0.825, and Factor 4: α = 0.864.

#### 2.3.3 Coping styles questionnaire

The Brief Coping to Problems Experienced Inventory (Brief COPE) was used to examine participants' coping styles. Initially developed by Carver (1997) and validated in Greek by Kapsou et al. (2010), the Brief COPE examines the cognitive and behavioral strategies people use to cope with challenges and stress. The scale consists of 28 items, rated on a Seven-point Likert-type scale ranging from 1 (I haven't been doing this at all) to 4 (I have been doing this a lot). The Brief COPE has been used in different factor structures in the literature. In the present study, both the adaptive-maladaptive (also referred to as approach-avoidant) framework (Carver, 1997; see also Eisenberg et al., 2012; Gloria and Steinhardt, 2016) and the three-factor model (Dias et al., 2012) were utilized. The adaptive-maladaptive framework distinguishes between two main coping strategies (adaptive and maladaptive) based on their effectiveness in managing stress. Adaptive coping strategies (i.e., planning, active coping, acceptance, positive reframing, emotional support, and instrumental support) refer to strategies that are generally considered effective ways of managing stress. In contrast, maladaptive coping strategies (i.e., denial, venting, self-distraction, substance use, self-blame, and behavioral disengagement) are generally considered less effective or even harmful in the long term. The three-factor model further classifies coping into three styles: problem-focused (i.e., active coping, use of instrumental support, positive reframing, and planning), emotion-focused (i.e., emotional support, venting, humor, acceptance, self-blame, and religion), and avoidance (i.e., self-distraction, denial, substance use, and behavioral disengagement). In this model, problem-focused and most emotion-focused strategies (with the exception of self-blame) are classified as adaptive strategies, whereas avoidance is considered a maladaptive coping strategy. The Brief COPE indicated satisfactory internal consistency for the current sample, with a Cronbach's alpha value of 0.760 for the overall scale. The reliability for the adaptive and maladaptive coping strategies was also acceptable with a Cronbach's alpha of 0.795 for adaptive coping and a Cronbach's alpha of 0.705 for maladaptive coping. For the three-factor model, reliability was also satisfactory; problem-focused: α = 0.759, emotion-focused: α = 0.638, and avoidance: α = 0.684.

### 2.4 Data processing and measures

After data collection, we proceeded with preliminary data processing and measures. The NMP-Q was used to extract an NMP-Q total score for each participant, categorizing their nomophobia into four levels: (a) absence of nomophobia, (b) mild nomophobia, (c) moderate nomophobia, and (d) severe nomophobia. Additionally, NMP-Q was used to calculate the four factors of nomophobia: (a) not being able to communicate, (b) losing connectedness, (c) not being able to access information, and (d) giving up convenience for each participant. To ensure that the factors align with those suggested by the questionnaire and measure the intended constructs, we conducted a factor analysis. The analysis confirmed the reliability of the NMP-Q and verified that the factors fit the questionnaire's four-construct structure. Specifically for the factor “Not being able to communicate,” the factor loadings ranged from 0.870 to 0.768. For the Factors “Giving up convenience,” “Not being able to access information,” and “Losing connectedness,” factor loadings ranged from 0.806 to 0.483, 0.878 to 0.572, and 0.841 to 0.565, respectively. The factor with the highest average among the four factors was “not being able to communicate” (M = 4.702, SD = 1.649), followed by “not being able to access information” (M = 4.693, SD = 1.357). Similarly, the Brief COPE was used to categorize participants into two coping strategies, (a) adaptive and (b) maladaptive, as well as to the three coping styles of the participants: (a) problem-focused, (b) emotion-focused, and (c) avoidance. Factor analysis was conducted to examine both the adaptive-maladaptive framework and the three-factor model (i.e., problem-focused, emotion-focused, and avoidance) of the Brief COPE. While the adaptive-maladaptive framework showed relatively clear item loadings (ranging from 0.646 to 0.256 for adaptive coping and 0.708 to 0.226 for maladaptive coping), the analysis of the three-factor model revealed some variation in item loadings. This variation likely reflects the conceptual overlap of coping strategies, which has been widely reported in coping literature (see Solberg et al., 2022, for a review). Despite this variation, the three-factor model aligned mainly with prior research that utilized the model (Dias et al., 2012), with factor loadings ranging from 0.723 to 0.286 for problem-focused coping, 0.836 to 0.206 for emotion-focused coping, and 0.600 to 0.363 for avoidance coping. Given the flexible nature of the Brief COPE (Carver, 1997) and its use across various models, we followed established practices in coping research and retained the items according to the conceptual framework chosen for this study.

Additionally, age and education categories were created to distribute our sample evenly for analytical purposes. Participants were divided into three groups: the emerging adults group included participants between the ages of 18–25 years old, the early-aged-adults group comprised participants aged between 26 and 39 years old, and the middle-and-late-aged adults group included participants between the ages of 40 and 77 years old. For education level, participants who completed their secondary education were classified in the low education group, those with a bachelor's degree in the middle education group, and those with a master's and/or doctorate degree in the high education group.

### 2.5 Statistical analysis

All data were entered into the Statistical Package for Social Sciences, Version 29.0 (SPSS 29, IBM Corporation, Armonk, NY, USA), and the significance level for the tests was set to 5%. Preliminary analyses included calculating descriptive statistics for the various demographic factors, the reasons for smartphone usage, and the prevalence of different types of nomophobia. For all the parametric tests, the assumption of normality was approximately satisfied. An independent sample *t*-test was used to examine if there were significant differences between the two genders and the level of nomophobia they exhibited. Univariate ANOVAs and Tukey HSD *post-hoc* tests were used to examine significant differences between age and nomophobia and education level and nomophobia. Crosstabs and chi-square tests also examined the significance between the different groups for each demographic factor and the different types of nomophobia. Smartphone usage and its relationship to nomophobia were examined with crosstabs and Pearson correlation analysis. Univariate ANOVAs and Tukey *post-hoc* tests were computed to investigate the associations between coping strategies and nomophobia.

## 3 Results

Hypothesis 1 was supported. There were high levels of nomophobia in the current sample. As Table 1 clearly shows, when participants are divided into four groups based on their total nomophobia score, only two participants fell within the “absence” level of nomophobia. Note that although the two participants with an absent level of nomophobia were included in the sample, they were excluded from the analyses that examined associations with nomophobia type, as the group size was considered small to conduct further tests.

**Table 1:** Distribution of nomophobia level in the current sample.

	**Absence**	**Mild**	**Moderate**	**Severe**
No.	2	70	154	74
Percent	0.7	23.3	51.3	24.7

To examine Hypothesis 2 and the statistically significant differences between nomophobia and age, a Univariate ANOVA with the nomophobia total score as the dependent variable and age groups (emerging adults, early-aged-adults, and middle-and-late-aged adults) as the independent variables was computed. Levene's test for equality of variances was not significant, *F*_(2, 297)_ = 2.443, *p* = 0.089, indicating that the assumption of homogeneity of variances was met. The analysis revealed statistically significant differences between nomophobia and age [*F*_(2, 297)_ = 5.616, *p* = 0.004]. Further, *post-hoc* analysis showed differences between emerging adults and middle-and-late-aged adults, with emerging adults exhibiting higher levels of nomophobia with a mean difference of 3.497 (*p* = 0.003). Further examination between the three types of nomophobia (excluding the type absence), a Chi-Square test showed that age was significantly related to the type of nomophobia χ^2^(4, *N* = 298) = 12.967, *p* = 0.011 and that there are significant differences among types of nomophobia for each age group. For the emerging adults group, mild nomophobia was found in 20% of participants, moderate nomophobia in 37.7% of the participants, and severe nomophobia in 41.9% of the participants. For the early-aged-adults, the percentages for each type of nomophobia were 28.6% for mild, 31.8% for moderate, and 28.4% for severe. For the middle-and-late-aged group, 51.4% of the participants had a mild level, 30.5% had a moderate level, and 29.7% had a severe level. Therefore, this part of Hypothesis 2 was supported.

To investigate statistically significant differences between nomophobia and gender, the category “other” was excluded from the analysis since there was only one participant who identified as other. An Independent Sample *T*-test was computed for 299 participants using the total score of nomophobia and gender. Levene's test for equality of variances was not significant, *F*_(297)_ = 0.035, *p* = 0.852, indicating that the assumption of homogeneity of variances was met. The analysis showed statistically significant differences between the 205 women (M = 84.33, SD = 24.87) and the 94 men [M = 71.06, SD = 25.38, *t*_(297)_ = 4.256, *p* < 0.001]. The Chi-Square test also showed that gender was significantly related to the type of nomophobia χ^2^(2, *N* = 297) = 23.912, *p* < 0.01. Further analysis using Crosstabs for the different levels of nomophobia (mild, moderate, severe) showed that among women, 15.7% exhibit mild nomophobia, 54.9% exhibit moderate nomophobia, and 29.4% exhibit severe nomophobia. For males, the percentages were 40.9%, 44.1%, and 15.1%, respectively. Thus, this part of Hypothesis 2 was also supported.

To examine possible statistically significant differences between nomophobia and education level, an ANOVA was conducted with nomophobia total score as the dependent variable and educational level (low, middle, high), as the independent variable. Levene's test for equality of variances was not significant, *F*_(2, 297)_ = 2.404, *p* = 0.092, indicating that the assumption of homogeneity of variances was met. The analysis revealed statistically significant differences between nomophobia and education level [*F*_(2, 297)_ = 4.479, *p* = 0.012]. *Post-hoc* analysis showed that the statistically significant differences were among the high education level group and the low education level group, with the latter exhibiting a higher level of nomophobia with a group mean difference of 1.59 (*p* = 0.016). Further Chi-Square test examining the type of nomophobia (mild, moderate, and severe) and education level showed no significant relationship χ^2^(4, *N* = 298) = 8.478, *p* = 0.076.

To examine Hypothesis 3 and possible associations between reasons for phone usage and nomophobia, results showed that the primary reason for phone use was communication, with participants spending an average of 4.70 h on this activity and 89.7% reporting that they use their phones for this purpose. Descriptive statistics showed that communication is the most common reason for phone use, with 27% of all multiple indicating communication as a reason for phone use. Social media followed closely, representing 26.4% of multiple responses. Information, professional purposes, games, and other reasons were less common, with percentages at 16.9%, 17.1%, 8.7%, and 3.9% respectively. Crosstabs analysis showed that most participants who used their phones for communication and social media exhibited moderate levels of nomophobia. Given that these were the most common reasons, further analysis was conducted to examine their correlation with nomophobia. Pearson correlation analysis showed a statistically significant positive correlation between communication and nomophobia [*r*_(300)_ = 0.624, *p* < 0.001], as well as between social media usage and nomophobia [*r*_(300)_ = 0.336, *p* < 0.001]. These results support the third hypothesis.

For Hypothesis 4 and the relationship between coping styles and nomophobia, a multinomial regression was used to see the effects on the type of nomophobia (i.e., mild, moderate, and severe) with respect to the three independent variables (problem-focused coping, emotion-focused coping, and avoidance coping). The reference category used was severe nomophobia. Please refer to Table 2. The overall model was statistically significant χ^2^(6, 298) = 57, 346, *p* < 0.001. The Nagelkerke *R*^2^ was 0.201 suggesting that 20% of the nomophobia could be explained by the model. The likelihood ratio test indicated that avoidance coping was the only significant predictor χ^2^(2, *N* = 298) = 48.592, *p* < 0.001 in distinguishing between the levels of nomophobia. Goodness of fit test indicated good fit of data [χ^2^(558, *N* = 298) = 560.521, *p* = 0.462]. For the comparison between mild nomophobia and the reference category severe nomophobia, problem-focused coping was not statistically associated with the likelihood of experiencing mild nomophobia (B = −0.083, *p* = 0.855). In addition, emotion-focused was not a statistically significant predictor (B = 0.645, *p* = 0.249). The variable avoidance coping was statistically significant (B = −3.061, *p* < 0.001) with an odds-ratio [Exp (B)] of 0.047, indicating that individuals engaging in avoidance behavioral coping strategies are less likely to report mild nomophobia compared to severe nomophobia. For the comparison between moderate nomophobia and the reference category severe nomophobia, problem-focused coping did not show a significant effect (B = −0.205, *p* = 0.583). The variable emotion-focused was not statistically significant (B = −0.012, *p* = 0.979). The variable avoidance coping was a statistically significant predictor (B = −1.159, *p* = 0.001) with an odds-ratio [Exp (B)] of 0.314, indicating that individuals engaging in avoidance behavioral coping strategies were less likely to report moderate nomophobia compared to severe nomophobia.

**Table 2:** Multinomial regression between coping styles and levels of nomophobia.

**Parameter estimates**									
Type of nomophobia^*^		**B**	**Std. error**	**Wald**	**df**	**Sig**.	**Exp(B)**	**95% confidence interval for Exp(B)**	
								**Lower bound**	**Upper bound**
Mild	Intercept	4.419	1.526	8.384	1	0.004			
	Problem-focused	−0.083	0.454	0.033	1	0.855	0.920	0.378	2.242
	Emotion-focused	0.645	0.559	1.328	1	0.249	1.905	0.636	5.704
	Avoidance	−3.061	0.495	38.243	1	<0.001	0.047	0.018	0.124
Moderate	Intercept	3.815	1.227	9.661	1	0.002			
	Problem-focused	−0.205	0.373	0.302	1	0.583	0.815	0.393	1.691
	Emotion-focused	−0.012	0.464	0.001	1	0.979	0.988	0.398	2.452
	Avoidance	−1.159	0.356	10.584	1	0.001	0.314	0.156	0.631

* The reference category is: Severe.

Another multinomial regression was used to see the effects of adaptive and maladaptive coping strategies on the level of nomophobia. The reference category was the severe level of nomophobia. The multinomial regression yielded significant results with χ^2^(4, *N* = 298) = 42, 317, *p* < 0.001. The Nagelkerke *R*^2^ was 0.152, suggesting that 15% of the nomophobia could be explained by the model. The likelihood ratio test indicated that the maladaptive coping strategy was the only significant predictor χ^2^(2, *N* = 298) = 38.803, *p* < 0.001 in distinguishing between the levels of nomophobia. Goodness of fit test indicated that the model adequately fits the data [χ^2^(436, *N* = 298) = 451.954, *p* = 0.289]. Adaptive coping strategy was not statistically associated with the likelihood of experiencing mild or moderate nomophobia (*p* > 0.05). Moreover, maladaptive coping strategy was statistically significant with (B = −0.235, *p* < 0.001) for mild and (B = −0.110, *p* < 0.001) for moderate. The odds ratio for maladaptive coping strategy indicated that for each unit of increase in maladaptive coping strategy, the odds of being in the mild or moderate category statistically decreased by 0.791 and 0.896, respectively. This means that individuals were less likely to have mild or moderate nomophobia, respectively, compared to the severe level category. A multiple regression was used to assess the effect of the four factors of nomophobia on adaptive coping strategy. The overall model was significant [*F*_(4, 293)_ = 10.221, *p* < 0.001] with *R*^2^ = 0.122 indicating that 12% of adaptiveness can be explained by the four factors of nomophobia. The results showed that losing connectedness (B = −0.334, *p* = 0.003) and giving up convenience (B = −0.092, *p* = 0.05) were statistically significant, with higher levels of losing connectedness and giving up convenience having lower levels of adaptiveness. In contrast, losing communication and not being able to access information did not have any statistically significant results (*p* > 0.05).

Another multiple regression was used to explore the relationship between maladaptive coping strategy and the four factors of nomophobia as previously defined. The assumptions of normality, linearity, independence, and homoscedasticity were approximately satisfied. Please refer to Table 3. The overall model was significant with *F*_(4, 293)_ = 15.691, *p* < 0.001, *R*^2^ = 0.176, indicating that 17.6% of maladaptiveness can be explained by the four factors of nomophobia. Statistically significant variables were the variables not being able to access information (B = 0.158, *p* = 0.047) and not being able to communicate (B = 0.151, *p* = 0.009), with higher levels of not being able to access information and not being able to communicate leading to higher levels of maladaptive coping strategy. The variables losing connectedness and giving up convenience did not have any statistically significant results (*p* > 0.05).

**Table 3:** Multinomial regression between coping strategies and levels of nomophobia.

**Parameter estimates**									
Type of nomophobia^*^		**B**	**Std. error**	**Wald**	**df**	**Sig**.	**Exp(B)**	**95% confidence interval for Exp(B)**	
								**Lower bound**	**Upper bound**
Mild	Intercept	3.770	1.455	6.714	1	0.010			
	Adaptive	0.039	0.024	2.681	1	0.102	1.039	0.992	1.089
	Maladaptive	−0.235	0.041	32.167	1	<0.001	0.791	0.729	0.858
Moderate	Intercept	3.728	1.200	9.650	1	0.002			
	Adaptive	−0.003	0.020	0.026	1	0.872	0.997	0.959	1.036
	Maladaptive	−0.110	0.032	12.033	1	<0.001	0.896	0.842	0.953

* The reference category is: Severe.

## 4 Discussion and conclusions

The current study adds to the growing body of research examining the impact of smartphones on mental health and particularly to the recent phenomenon of nomophobia. Smartphones, often considered one of the most significant “addictions” of the twenty-first century (Santl et al., 2022), are multifunctional devices that offer a wide range of applications (Vagka et al., 2023b) for convenience, communication, connectedness, and overall ease with everyday tasks, making them an essential part of daily life, especially for future generations. Although this variety of uses benefits users in multiple ways, the adverse outcomes of excessive smartphone use should not be ignored (Kang and Jung, 2014). The current study aimed to explore the prevalence of nomophobia among adults in Cyprus and its relationship with demographic characteristics, phone usage, and coping styles. Overall, the current results suggest that most individuals in Cyprus exhibit some level of nomophobia. Additionally, younger adults were found to be more prone to nomophobia than older adults, women exhibited higher levels of nomophobia than men, and education appeared to play a role in someone's development of nomophobia. Furthermore, maladaptive coping styles and avoidance strategies were identified as possible factors influencing the severity of nomophobia. The current study's findings contribute to the existing literature examining the factors influencing this emerging phenomenon.

First, our results show that the vast majority of our sample (99.3%) shows some signs of nomophobia, with only two participants showing no signs. Significantly, 76% of the participants exhibited at least moderate fear or anxiety related to being without their smartphones. This indicates that nomophobia is of great concern and affects a large portion of the population of Cyprus. This finding supports our first hypothesis that predicted a high prevalence of nomophobia in the current sample and aligns with the existing literature that suggests the growing problem of nomophobia (Al-Mamun et al., 2023; Anjana et al., 2021; Ferchichi et al., 2023; Kaviani et al., 2020; Oraison and Wilson, 2024; Saleh et al., 2019; Santl et al., 2022). In particular, our findings align with those of Greece (Gnardellis et al., 2023b) which indicated similar percentages in this population (75.9%). Importantly, however, the number of participants with severe nomophobia was higher in Cyprus compared to Greece (24.67% in Cyprus vs. 18.9% in Greece), indicating that the intensity of nomophobia may be more pronounced in certain regions. This is supported by findings from other countries, such as Italy, that found lower levels of nomophobia (Bragazzi et al., 2019). A possible explanation for the finding that Cyprus has high levels of nomophobia could be the increasing smartphone usage in Cyprus, which, as previously mentioned, exceeds the EU average (CyprusMail, 2021). Our results also suggest that the main factor influencing nomophobia is the lack of communication, followed closely by access to information. Therefore, it appears that for the current sample, the primary driver of their anxiety or fear is the potential loss of their ability to use their phones for communication and obtaining information they might need. On the contrary, connectedness and convenience do not appear to influence our participants significantly. This is a very interesting finding because it highlights that, at least in the current sample, losing essential functions like communication and access to information may be more central to developing nomophobia than more general benefits of connectivity or convenience. It would be valuable for future research to further explore this finding for a more thorough understanding of the reasons behind reliance on smartphones.

Second, the current results show that demographics play a role in the development of nomophobia. Looking at age, the current results show that younger individuals are more prone to developing nomophobia, aligning existing literature (Guimarães et al., 2022; Guzel and Alan, 2020; Kaviani et al., 2020; Vagka et al., 2023b), while contrasting findings that suggest that age does not play a role (Adawi et al., 2018; Galhardo et al., 2020; Saleh et al., 2019). Specifically, our results indicate that emerging adults exhibited higher levels of nomophobia, with 41.9% displaying severe symptoms, compared to 29.7% in middle-to-late-aged adults. In contrast, over half of the older age group had mild nomophobia. At the same time, only 20% of emerging adults fell into this category, which supports that younger adults are more likely to experience severe nomophobia. This is a significant finding, as it highlights that younger adults are more vulnerable to the adverse effects of smartphone usage, like fear, anxiety, or dependence. Understanding this trend is of significant importance for future research. As younger individuals increasingly integrate smartphones into their daily lives, it becomes essential to identify strategies to protect them from potential mental health concerns, including the development of severe nomophobia. Looking at gender differences, the current results align with studies that indicate that women are more likely to experience higher levels of nomophobia compared to men (Galhardo et al., 2020; Kaviani et al., 2020; Moreno-Guerrero et al., 2020; Vagka et al., 2023b). The results reveal that women were nearly twice as likely to exhibit severe nomophobia. Notably, among individuals exhibiting severe nomophobia, 81.1% are women, while men are more likely to exhibit milder levels. This suggests that women may be more vulnerable to the harmful effects of smartphone dependence, highlighting the need for further research into the psychological and social factors contributing to these gender differences. Understanding these underlying causes could help refine interventions and develop more targeted strategies to address smartphone overuse in women. The current study also suggests associations between nomophobia and education level. Since the connection between education and problematic smartphone use has not been studied in the past, the current results provide an initial exploration of this relationship. Our findings suggest that participants with lower education levels tend to exhibit higher levels of nomophobia compared to those with higher education levels. However, this finding should be interpreted cautiously, as no significant relationships were found when looking at specific types of nomophobia. A possible explanation for this finding is the sample size, as the statistical power increases when the “absent” nomophobia category is removed. Given the lack of existing literature on the relationship between education level and nomophobia, further investigation is recommended.

Third, the current results contribute to the growing literature that suggests a connection between social media use and nomophobia (Jeong et al., 2016; Okur et al., 2022; Vagka et al., 2024). The current results show that the primary reasons individuals use their phones are for communicating and social media. Our analysis revealed that those who use their phones for these purposes are more likely to exhibit moderate levels of nomophobia. Additionally, our data suggest a clear link between nomophobia and the amount of time spent on communication and social media, indicating that increased use in these areas is associated with higher levels of nomophobia. This relationship needs further exploration in future studies.

Lastly, a consistent positive relationship was found between nomophobia and avoidant coping strategies across all types of nomophobia. That is, individuals with higher levels of nomophobia are more likely to rely on avoidant coping mechanisms, such as behavioral disengagement or denial, rather than facing the situation directly. This suggests that as nomophobia increases, so does the tendency to use less effective, avoidant strategies to cope with the fear of being without a smartphone, which aligns with previous research findings (Lu et al., 2021). Similarly, a positive relationship was found between nomophobia and maladaptive strategies, with those exhibiting higher levels of nomophobia being more likely to rely on maladaptive coping. Specifically, 76.5% of participants who adopted maladaptive coping strategies displayed moderate to severe levels of nomophobia, a finding supported in previous literature (Bragazzi et al., 2019). On the contrary, our results show a negative relationship between nomophobia and problem-focused coping strategies when looking at the overall nomophobia score, suggesting that individuals who employ a greater extent of problem-solving strategies tend to experience lower levels of nomophobia. However, when examining specific types of nomophobia, this relationship was not significant. Notably, the result approached significance when participants with no nomophobia were excluded, indicating that the link between problem-focused coping and nomophobia remains somewhat unclear and open for future investigations with a larger sample size. Finally, the current results suggest no relationship between emotion-focused coping and nomophobia, which aligns with findings from previous research (Lu et al., 2021).

The findings of the current study are particularly significant given the broader mental health challenges that the population of Cyprus is experiencing. According to the European Commission's 2023 Health Profile, 7.2% of the population experiences anxiety disorders, and 3.85 suffer from depressive disorders (European Observatory on Health Systems and Policies, 2023). The high prevalence of nomophobia identified in this study may contribute to these existing mental health challenges, especially amongst the youngest population who heavily relies on their smartphones. These findings emphasize the need for targeted interventions to reduce smartphone dependence and its negative mental health impact. Practical implications of this study include developing educational programs that promote healthier smartphone usage, especially for younger adults who are most vulnerable. Additionally, mental health professionals could benefit from integrating assessment of smartphone use into routine screening, as excessive use may be a contributing factor to nomophobia, fear, or anxiety. Moreover, these results could be used in public health initiatives aiming at mitigating the psychological risk associated with smartphone overuse. It is important for future research to explore these relationships further.

Though the current study provides valuable insights regarding nomophobia, it is not without limitations. First, the study examined associations between variables rather than causal relationships, meaning it cannot determine whether nomophobia is a cause or effect of the examined factors. Second, the generalizability of the current findings should be approached cautiously due to the small sample size and the use of the convenience sampling method. This sampling method also did not allow us to distinguish whether participants completed the survey only once. Additionally, most participants were female, possibly introducing an overrepresentation of this population and thus introducing bias. Also, the study was limited in scope, as it focused only on demographics and coping styles, while other potentially relevant factors related to nomophobia were not examined. Lastly, the reliance on self-report measures should be viewed with discretion. Future research should aim for a more balanced gender distribution to gain a deeper understanding of potential gender differences. Moreover, other mental health issues, like loneliness, which may drive individuals to rely more heavily on their phones and thus be more prone to nomophobia (Valenti et al., 2022; Zhang et al., 2023) were not investigated in this study and should be the focus of future research. Beyond these factors, future research with bigger and more diverse samples should aim to confirm the structure of coping strategies, especially given the observed overlap of coping strategies reported in the literature. Future studies could also explore additional psychological, contextual, or behavioral factors that may contribute to a more comprehensive understanding of nomophobia and its impact on individuals.

In conclusion, this study indicates that nomophobia is undeniably spreading at a rate that should not be ignored, highlighting the urgent need for action. While smartphones offer numerous benefits, their excessive use can negatively impact mental health. This study adds valuable insights into the growing body of research on nomophobia and its impact on mental health in Cyprus, where smartphones have become an integral part of daily life. Our findings highlight that nomophobia is highly prevalent among the population, particularly among younger adults and individuals with lower education levels. The results of the current study also show that maladaptive coping and avoidant strategies contribute to the severity of nomophobia, raising concerns about its potential impact on mental health. Given the broader mental health challenges in Cyprus, where a considerable portion of the population faces anxiety disorders and depression, this study underscores the need for further research and targeted interventions. Future studies are urgently needed to explore additional variables, such as loneliness, to provide a more comprehensive understanding of this phenomenon. The findings of this study can be used by professionals in their efforts to tackle this growing issue. The development of educational programs promoting healthier smartphone habits and integrating smartphone use assessments into routine health care screenings is highly recommended.

## Data Availability

The raw data supporting the conclusions of this article will be made available by the authors, without undue reservation.
